# Scaffolding Simulation Activities for Medical Students Learning Cardiopulmonary Assessment: A Retrospective Study

**DOI:** 10.7759/cureus.82013

**Published:** 2025-04-10

**Authors:** Timothy J Hodge, Laura J Potter, Carter J Helsby

**Affiliations:** 1 Simulation, Liberty University College of Osteopathic Medicine, Lynchburg, USA

**Keywords:** curricular design, medical education, medical simulation, scaffolding, zone of proximal development

## Abstract

The purpose of this study was to determine if the scaffolding of simulation activities and modalities improved medical student outcomes as part of a cardiopulmonary course at the researchers’ medical school. The concept of scaffolding has been used in educational curricular design for many years, but its application in healthcare simulation has been poorly studied. As a new medical school, the order in which the simulation educational events were planned had largely been based on anecdotal and observational evidence from faculty. Additionally, there had been great variability in the modalities used during simulation learning activities during the initial curriculum development. The current study sought to quantify whether the current order of scaffolded simulation activities that started in 2020 had statistically increased the summative simulation scores compared to those prior to 2020. The findings suggested there was an increase of 3% in student scores found via an independent samples t-test, t(1287) = -12.252, p < .001. While the researchers are unable to suggest that the scaffolded activities were linked with the causation of the increase in scores, the order of simulation activities could be contributory. These findings suggest scaffolding of simulation activities could influence student learning in a similar manner to other educational activities. Future research should seek to better understand the role of scaffolding in medical simulation.

## Introduction

The use of scaffolding in education has been a long-used theory to provide learners the ability to increase their self-efficacy over time [1]. Wood et al. have put forth that “instructional scaffolding enables a…novice to solve a problem, carry out a task, or achieve a goal that he or she cannot accomplish on his or her own [2] and describes a support that can easily be removed when no longer needed. A key feature of scaffolding is fading the support provided to the learner over time” [3] as the learner gains control of their own learning. Effective scaffolding methodically takes simple tasks and increases their complexity over time while slowly removing instructional support, ultimately culminating in building to their assimilation into very complex tasks. In this way, the ideal state would provide the instructional support necessary to permit a student to increase their learning in this manner, yet promote critical thinking.

Medical education and curriculum design generally follow the same concept of scaffolding. Cognitive load theory fits into this thinking and is often cited in the literature in designing medical curricula [4]. Three issues are considered in the cognitive load theory: task fidelity, task complexity, and instructional support [4]. This is directly applicable to medical simulation, where simulation activities are chosen based on the same three concepts. Learners thus work from high-support, low-complexity, low-fidelity (e.g., labs with preceptors guiding practice on partners or task trainers) to low-support, high-complexity, high-fidelity circumstances (e.g., Objective Structured Clinical Examination (OSCE)) [4]. While this is purposeful in most cases, the endeavor may be indirect. Such an example was the origin of this research project proposed by the authors.

In this study, the authors were involved in curricula revisions of various simulation activities within the cardiopulmonary course in a medical school based on anecdotal evidence of their efficacy. In the curriculum development process of how to train medical students on both heart sounds and lung sounds, the educators experimented with a rudimentary method of trial and error to establish the best order and formatting of the simulation activities during the course, varying in support, complexity, and fidelity. In 2020, the educators and course director of the program put together the order of activities that would be utilized to the current time. This ultimately provided a scaffolded approach to the simulation education activities, but it was based on the group’s feelings anecdotally that students were performing better and meeting the learning objectives of the course at a higher level. However, this had not been demonstrated through the analysis of summative assessments or other quantitative data.

## Materials and methods

The purpose of this quantitative, retrospective, cohort study was to determine if the order (i.e., scaffolding) of simulated training events significantly improved outcomes for medical students learning cardiopulmonary assessment. The study investigated whether the order of simulated events between 2015 and 2023 for the second-year cardiopulmonary course led to better student outcomes as measured by students’ final cardiopulmonary standardized patient (SP) OSCE scores. Students were grouped into cohorts of complete second-year classes, years 2015-2019 and years 2020-2023, reflecting the implementation of our current scaffolding model in 2020 (Table 1).

**Table 1:** Order of Simulation Events for Cardiopulmonary Assessment CP: Cardiopulmonary; OSCE: Objective Structured Clinical Examination; SP: Standardized Patient; Sim: Simulation The Columns Represent the Order of Activities by the Week Within Each Academic Year. The Rows Reflect the Specific Learning Activities.

Year	2015	2016	2017	2018	2019	2020	2021	2022	2023
Week 1	Cardiac Exam	Cardiac Lab With Heart Sounds	Cardiac Lab With Heart Sounds	Cardiac Lab With Heart Sounds	Cardiac Lab With Heart Sounds	Cardiac Lab With Heart Sounds	Cardiac Lab With Heart Sounds	Cardiac Lab With Heart Sounds	Cardiac Lab With Heart Sounds
Week 2	Cardiac Sim	Harvey Heart Sounds Lab	Harvey Heart Sounds Lab	Harvey Heart Sounds Lab	CP Sim	Cardiac Lab With Heart Sounds With SP	Cardiac Lab With Heart Sounds With SP	Cardiac Lab With Heart Sounds With SP	Cardiac Lab With Heart Sounds With SP
Week 3	Fall Break	Fall Break	CP Sim	CP Sim	Fall Break	CP Sim	CP Sim	CP Sim	CP Sim
Week 4	Pulmonary Chest X-Ray Lab	Pulmonary Lab	Fall Break	Fall Break	CP OSCE	Fall Break	Fall Break	Fall Break	Fall Break
Week 5	Pulmonary OSCE	CP OSCE	CP OSCE	CP OSCE	N/A	CP OSCE	CP OSCE	CP OSCE	CP OSCE
Week 6	N/A	CP Simulation	N/A	N/A	N/A	N/A	N/A	N/A	N/A

Research question 

The research question is as follows: Is there a difference in student cardiopulmonary OSCE scores between students who participated in simulation events before and after 2020, when the current scaffolded model was developed?

Hypothesis

The null hypothesis is as follows: there is no significant difference in student cardiopulmonary OSCE scores between students who participated in simulation events before and after 2020, when the current scaffolded model was developed, as measured by the students’ composite OSCE score.

Procedure and data analysis

The researchers sought approval from the Institutional Review Board (IRB) of Liberty University, Lynchburg, VA, to conduct the research and were granted permission to proceed as an exempt study (approval number: IRB-FY24-25-85 dated July 22, 2024). The data utilized within this study are limited to reporting of only the OSCE test scores and contain no identifiable information that could be traced back to anyone. The data consisted of the year in which the score was obtained and the composite percentage score. The summative OSCE assessment was held at or near the end of the cardiopulmonary course, and the composite score for this assessment consisted of three components. The OSCE was structured as a simulated patient office visit where the learner was provided a brief description of why the patient was visiting the simulated medical office on that date. The student would then enter the exam room to perform an interview of the patient and conduct an appropriate physical exam. Following the patient encounter, the student would exit the room and summarize the encounter in written form. The graded components of the assessment were the performance of the student’s written patient note, performance on the physical exam, and a humanistic assessment of the student by the SP during the encounter. The simulation activities that were scaffolded throughout the course taught different elements of all these scored items. The OSCE provides an overall assessment of the students’ performance during the course and could be viewed as a direct evaluation of the effectiveness of the simulation activities.

After analysis and identification of the scaffolding of simulation instructional events for the course for every year, it was determined that the current model of scaffolding was established during the calendar year 2020. Therefore, the analysis of the data consisted of the pre- and post-2020 cardiopulmonary course scaffolding for simulation events. For clarification’s sake, the current scaffolding consists of students participating in a cardiopulmonary exam lab, a cardiopulmonary exam lab with a SP, the cardiopulmonary simulation, fall break, and the cardiopulmonary OSCE. Previously, the scaffolding was inconsistent between the years, with different simulation activities falling on different weeks throughout the semester. The dependent variable, which was the cardiopulmonary OSCE scores, was pulled from the program’s simulation learning management system. A report was generated to pull a student’s composite score as a percentage. The percentage was converted to a numerical decimal figure to run the analysis. The report permitted dividing scores by year, which ran from 2015 until 2023. All the scores were part of the second-year cardiopulmonary course OSCE assessment and subject to only the variability found within each cohort of students within the school. Variability within each student group's sex, age, or skill level would be minimal with only the school’s admissions criteria or matriculation rates contributing to any minor inconsistencies in each annual cohort.

The learning management system where the data is maintained is restricted to a limited number of faculty and staff via university computing sign-on credentials. After the data was downloaded, it was stored in a university SharePoint folder (Microsoft Corp., Redmond, WA) that was only accessible to the researchers. The data will be retained for a period of at least seven years and will be deleted thereafter.

The report generated a total of 1,362 student scores. Only the scores and the year of the scores were retained, with no identifiable personal data pulled from the report. Because all students who scored less than 0.795 would have had an opportunity to retake the exam a second time, which would result in two scores and possible improvement or decline in scores for only some students, all first-time failure scores were removed to produce a replicable set of data for each year. Additionally, since failures account for a small percentage of the scores, these data points would have likely been extreme outliers and obscured the results. After this data transformation, the total number of student scores was 1289. The data was labeled with a “0” if the score was before 2020 and with a “1” if the score was between 2020 and 2023.

Since the researchers sought to determine if a statistical difference existed between the two groups, the data analysis was performed utilizing an independent-samples t-test using IBM SPSS Statistics software, version 29.0 (IBM Corp., Armonk, NY). To determine the suitability for this type of test, the researchers looked at the six assumptions of an independent-samples t-test [5]. The first assumption was met by one dependent variable was a continuous variable, which was the cardiopulmonary OSCE scores [5]. The second assumption was met by one independent variable that consisted of two categorical, independent groups, which were the pre- and post-2020 time periods when students participated in the scored OSCE [5]. The third assumption was met because there was independence of observations with no relationship between the observations in each group [5]. Each class was independent and consisted of a new cohort of students each year. This was considered valid despite the potential of a few students participating more than once because of a failure to matriculate the previous year. The fourth assumption found there were five significant outliers in the data set, but because the sample size was so large, which would likely not overwhelmingly change the results, the researchers decided to keep these scores [5]. The fifth assumption showed that cardiopulmonary OSCE scores for both pre- and post-2020 time periods were normally distributed as assessed by visual inspection of normal Q-Q plots. There was some conflict with this inspection noted with a pre-2020 skewness of .098 (SE = .093) and a kurtosis of -.838 (SE = .186) and a post-2020 skewness of -.571 (SE = .100) and a kurtosis of -.262 (SE = .199). To further explore this assumption, a Kolmogorov-Smirnov (KS) test of normality showed pre-scaffolding data D(688) = .05, p < .001, and post-scaffolding data D(601) = .08, p < .001. These results suggest the data is not distributed normally. Despite these results, the researchers acknowledged the non-normality of the data and chose to proceed because non-normality does not substantially affect the Type I error rate, and independent-sample t-tests are considered robust (Laerd Statistics, 2015, Lund Research Ltd, Derby, UK). Additionally, since the sample size is very large with small D values for the KS results suggesting mild non-normality, no variance issues via the Levene’s test, and a very high statistical power, the t-test’s robustness is sufficient for analysis. The sixth assumption showed there was homogeneity of variances for cardiopulmonary OSCE scores for pre- and post-2020 time periods, as assessed by Levene’s test for equality of variances (p = .097) [5].

## Results

The researchers found a statistically significant difference in the mean cardiopulmonary OSCE scores between pre- and post-2020 time periods, t(1287) = -12.252, p < .001. There was a statistically significant difference with pre-2020 (M = .89, SD = .05) students scoring -.03, 95% [-.04 to -.03] lower than post-2020 (M = .92, SD = .05) mean cardiopulmonary OSCE scores. These results demonstrated that students scored three percentage points higher in their OSCEs under the current simulation scaffolding model versus those students under different scaffolding models prior to 2020 (Table 2). The effect size, as measured by Cohen’s d, was d = 0.05, indicating a very small effect, with a power of .95, conducted via G-Power (version 3.1.9.7, Heinrich-Heine-Universität Düsseldorf, Düsseldorf, Germany), at a significance level of .05. Because there was a statistically significant difference between the means (p <.001), the researchers rejected the null hypothesis and accepted the alternative hypothesis.

**Table 2:** Descriptive Statistics OSCE: Objective Structured Clinical Examination

PrePost		Cases					
		Valid		Missing		Total	
		N	Percentage	N	Percentage	N	Percentage
OSCE Score	Pre-scaffolding	688	92.3%	57	7.7%	745	100.0%
	Post-scaffolding	601	97.4%	16	2.6%	617	100.0%

**Figure 1:**
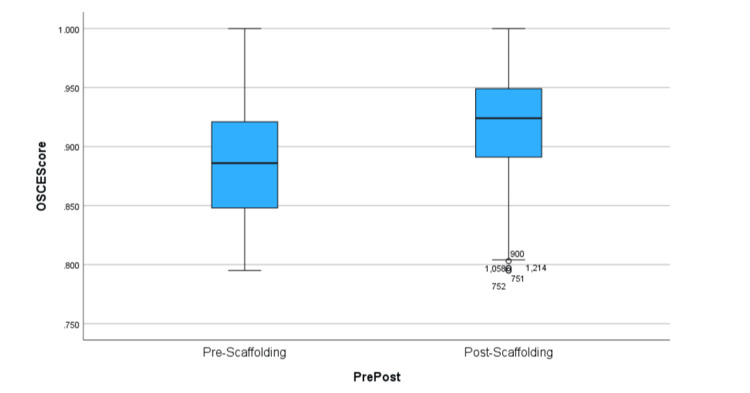
Box Plot of Pre-2020 and Post-2020 OSCE Scores OSCE: Objective Structured Clinical Examination The X-axis Shows the Box Plots of the Pre-2020 and Post-2020 Test Scores; the Y-axis Is the Actual Recorded Scores Along the Box Plot.

The researchers acknowledge that due to the large number of confounding variables likely encountered in our study, the potential for replication may be challenging. These confounders also present the challenge of suggesting that the scaffolding of simulation activities alone was a causality of these results. However, the researchers are not aware of any other significant curricular or learning techniques introduced or changes made during the study period. The box plot demonstrates both a change in the average score and a narrowing of the interquartile range (Figure 1). Scores appear to have increased in the period after 2020, and the variability of scores around the average decreased with the score change. This suggests not only did students score higher with the current scaffolding model, but they also scored higher more consistently. These results demonstrate the potential of greater student understanding and application of the cardiopulmonary course objectives as measured by the OSCE summative assessment. At a minimum, the results suggested that how the simulation activities were scaffolded for student learning was one of the variables contributing to better student scores. Since the researchers were unable to find similar studies suggesting that scaffolding simulation activity is effective, this study contributes to a starting point for further study.

## Discussion

Literature review

There are several nuances in defining scaffolding in medical education. Historically, scaffolding has described how a structure of support by the teacher to the student can be removed over time. This enables the student/novice learner to solve problems with help before they would have been able to solve them on their own [2]. Scaffolding interacts closely with the Zone of Proximal Development theory developed in the early 1960s. The Zone of Proximal Development can be described as the distance between what level of problem-solving a novice can perform independently versus the level they can perform at with the guidance of experts and more capable peers [6]. Students can often experience adverse effects in their learning due to excessive task complexity. Scaffolding the design and delivery of content allows learners to counteract those adverse effects. One key feature of scaffolding is the decrease of support to the learner over time, allowing the learner to be in control and take responsibility for their learning [3]. Also pertinent to the concept of scaffolding is the cognitive load theory, as noted above [4]. Its three relevant issues of task fidelity, task complexity, and instructional support are key to designing medical simulation activities.

Health science departments around the world utilize scaffolding in curriculum and content design and delivery. The study of scaffolding use in health science simulations is slim. These authors found no direct quantitative studies on scaffolding in medical education and outcomes. However, there are multiple examples of how scaffolding is effective in other domains, such as game-based learning, where both normal and dark play are utilized [7]. Medical education can be scaffolded by creating a curriculum where the learners gradually receive less and less support until they can solve specific problems on their own. The intention of scaffolding in this context is to progressively facilitate the integration of technical skills and medical knowledge. Much of the andragogy of medical education is based on this integration and progression into the complexity of patient care.

In this study, simulation is used as a medium to bring classroom learning into practical application. Medical simulation is an opportunity for students to work collaboratively with peers while performing a task with faculty support and feedback before they are required to perform similar tasks by themselves on patients or patient actors. Scaffolding has been utilized in this specific study by ordering how learners move from formative, team-based learning activities (i.e., high-fidelity simulations) to summative, case-based learning activities (i.e., OSCEs) [8]. The outcome of this scaffolding can be measured quantitatively through student performance. This study was a result of the literature calling for further investigation into scaffolding in “different settings such as ...simulation training” [7] and “the optimal transitions of different types of scaffolding with increasing levels of complex skills” [1]. More recently, it has been observed that “health sciences programmes need multifaceted, systematic, and programmatic approaches to scaffolding that begin with meticulous planning, design, and sequencing of curricula to build up necessary learning experiences for students” [9].

The findings of this study suggest that there is a particular order of scaffolding that is more beneficial for improving student outcomes. Future curricular development, specifically around simulation-based medical education, should focus on a thoughtful method of scaffolding. While this study shows one example of better student outcomes from scaffolding, other methods should be explored and studied to determine which method provides the greatest learning opportunities for the students.

Limitations 

The researchers acknowledge the significance of utilizing a dividing date between the groups in 2020 due to the pandemic and large changes occurring at every level of education during this time. While our results may be influenced by those events, the data covers a time both well before and after the pandemic. The researchers do not believe the adoption of the current model for teaching the cardiopulmonary course was directly related to changes in curriculum due to the pandemic, but was related to the regular evolution of instituting the best andragogy for student learning. 

The researchers also recognize there is the potential for confirmation bias that may result from this retrospective research. This study originated from the perspective that the simulation activities of the cardiopulmonary course had seemingly improved student outcomes, but there were no quantitative or qualitative data supporting these claims. To alleviate or lessen any potential confirmation bias, the researchers only ran the data a single time based on already established parameters and chose not to utilize any additional post hoc testing.

Suggestions for future research

Previous research has shown the benefit of scaffolding in learning complex skills within medical education. However, there is a paucity of evidence demonstrating the use of scaffolding specifically using simulation. This study suggests that scaffolding of simulation activities potentially benefits learner outcomes. Therefore, this research provides a novel opportunity to contribute to the body of knowledge in medical simulation.

While it could be perceived as a potential limitation to the study, there was only a single point of quantitative data available throughout the study period. Future research would benefit from exploring the measurement of student outcomes from multiple points of reference and potentially across different courses that employ similar methods of simulation. For example, the current study was built upon small groups with a task trainer and continued through individual evaluation with a standardized patient. Replication studies in either different systems or across other healthcare training areas may reinforce the benefit of correctly scaffolding simulation events to improve student outcomes.

## Conclusions

The data reviewed in this study are not conclusive in determining that a particular scaffolding methodology was solely responsible for increasing student scores, but because scaffolding has been shown to assist students in medical education, it was at least a potential contributory item. The informal method for initially putting together the way simulation events were scaffolded through the process of numerous discussions likely had some unsubstantiated validity because of the broad observations noted by the faculty and staff evaluating the medical students throughout the period studied. Therefore, while the authors do not claim the state of causation, it is insightful to determine that scaffolding in simulation-based educational activities has the potential to increase learning and the betterment of student outcomes in summative assessments.

The acquisition of mastery in accurately identifying pathologic heart or lung sounds is an important skill for physicians to develop. Therefore, an argument could be made that merely the repetition of the current scaffolded simulation model in hearing these sounds led to greater identification of the various murmurs and abnormal heart and lung sounds. However, there is the understanding that the summative OSCE assessment used as the measurement in this study is not solely scored on identification of the correct sound. Students correctly learning to both perform a proper cardiopulmonary assessment and document such an assessment are significant factors in their composite score. This is part of the learning objectives of the simulation activities. The scaffolded activities utilize the transition from team-based to case-based learning to reinforce the holistic approach towards becoming more proficient in a cardiopulmonary exam. The findings of this study are significant since they lend knowledge towards a more comprehensive scaffolding model within a cardiopulmonary course. Other courses within medical education could be scaffolded in a similar way to increase student learning.
